# The *Eupentacta fraudatrix* transcriptome provides insights into regulation of cell transdifferentiation

**DOI:** 10.1038/s41598-020-58470-0

**Published:** 2020-01-30

**Authors:** Alexey V. Boyko, Alexander S. Girich, Ekaterina S. Tkacheva, Igor Yu. Dolmatov

**Affiliations:** 1 National Scientific Center of Marine Biology, Far Eastern Branch, Russian Academy of Sciences, Palchevsky 17, 690041 Vladivostok, Russia; 2 Far Eastern Federal University, Suhanova 8, Vladivostok, 690950 Russia

**Keywords:** Gene ontology, Sequence annotation, Transdifferentiation

## Abstract

The holothurian *Eupentacta fraudatrix* is a unique organism for studying regeneration mechanisms. Moreover, *E. fraudatrix* can quickly restore parts of its body and entire organ systems, yet at the moment, there is no data on the participation of stem cells in the process. To the contrary, it has been repeatedly confirmed that this process is only due to the transformation of terminally differentiated cells. In this study, we examine changes in gene expression during gut regeneration of the holothurian *E. fraudatrix*. Transcriptomes of intestinal anlage of the three stages of regeneration, as well as the normal gut, were sequenced with an Illumina sequencer (San Diego, CA, USA). We identified 14,617 sea urchin protein homologs, of which 308 were transcription factors. After analysing the dynamics of gene expression during regeneration and the map of biological processes in which they participate, we identified 11 factors: Ef-EGR1, Ef-ELF, Ef-GATA3, Ef-ID2, Ef-KLF1/2/4, Ef-MSC, Ef-PCGF2, Ef-PRDM9, Ef-SNAI2, Ef-TBX20, and Ef-TCF24. With the exception of TCF24, they are all involved in the regeneration, development, epithelial-mesenchymal transition, and immune response in other animals. We suggest that these transcription factors may also be involved in the transdifferentiation of coelomic epithelial cells into enterocytes in holothurians.

## Introduction

Regeneration is a unique phenomenon in which the origin, evolution, and mechanisms are still poorly understood. In particular, stem cells are currently considered the main cellular source for most animals^1^. At the same time, even in the most primitive multicellular animals, such as sponges, regeneration can occur not only with the involvement of stem cells (archeocytes), but through the dedifferentiation of specialized cells, choanocytes^2,3^. In humans, with damage to the liver, lungs, and pancreas, specialized cells of these organs can dedifferentiate, entering the mitotic cycle, and participating in their repair^4^.

In echinoderms, regeneration apparently occurs through dedifferentiation or transdifferentiation of specialized cells, which has been confirmed by many studies^5–9^. There is, however, no direct evidence of the presence of stem cells in echinoderms or their involvement in morphogenesis^10^. In addition, there has recently been data that regeneration in these animals can occur with complete suppression of mitotic activity^9,11^, which indicates participation of specialized cells of organ remnants, rather than stem cells. In connection with this, and the ability to regenerate, echinoderms are convenient model objects for studying the involvement of differentiated cells in regeneration.

Among echinoderms, holothurians are capable of restoring almost all organs and tissue types, and as such are of special interest^5,12,13^. In addition, their unique feature is the ability to eject (eviscerate) their intestine in response to stressful stimuli; the lost part of the digestive tract regenerates by formation of two anlagen on the anterior and posterior parts of the body, which grow towards each other^14^. This process in most studied species of holothurians occurs as a function of the cells of luminal epithelia of the cloaca (posterior anlage) and the remaining part of the esophagus (anterior anlage), which is due to dedifferentiation, migration, proliferation, and differentiation^15–18^.

Gut regeneration mechanisms in *E. fraudatrix* are even more unusual. In this species, evisceration occurs through the anterior part of the body, which is lost (the pharynx and esophagus) with the entire digestive system^19–21^. In this situation, the anterior part of the intestine is formed with cells from the coelomic epithelium, as a derivative of the mesoderm^7^. Coelomic epithelium in echinoderms mainly consists of peritoneocytes and myoepithelial cells^22^, and contains neurons and bundles of axons that form the basiepithelial nerve plexus. After evisceration, the peritoneal and myoepithelial cells of the coelomic epithelium of gut mesentery, as well as of the anterior part of the animal, dedifferentiate. This is manifested as a loss of specialized structures, e.g., bundles of tonofilaments (peritoneocytes) and myofilaments (myoepithelial cells). Myofilaments are assembled into specific spindle-like structures and gradually degrade in the cytoplasm, or are ejected into the intercellular space^8^. At the same time, dedifferentiated cells of the coelomic epithelium migrate to the damaged edge of the mesentery, forming a cluster of flattened epithelial cells; neurons and nerve processes are probably destroyed. In the process of regeneration, part of the dedifferentiated cells of the coelomic epithelium undergo transdifferentiation, mitotically divide, and transform into enterocytes^7^.

Until recently, *E. fraudatrix* was the only species for which transdifferentiation of mesodermal cells into enterocytes was reported^7^. Recently, the ability of muscle cells to transform into enterocytes has been found in zebrafish^23^. It is not yet clear how similar the mechanisms of transdifferentiation in holothurians and fish actually are. One of the key genes that trigger transdifferentiation in zebrafish is *oct4*^23^. The holothurian ortholog of this gene, *oct-1/2/11*, does not show noticeable expression during intestinal regeneration in *Holothuria glaberrima*^24^. Moreover, the molecular basis of cell transdifferentiation mechanisms in *E. fraudatrix* remains unknown. In order to understand, to a first approximation, the possible molecular processes that occur during transdifferentiation in holothurians, we decided to use RNA-seq. In this work, we tried to obtain the complete transcriptome and determine the differential expressed genes (DEGs) at the stage of active cell transdifferentiation and transcription factors (TFs), which are candidates for the role of transdifferentiation regulator.

## Results and Discussion

### Formation of digestive epithelium by coelomic epithelial cells

The morphological features of regeneration of internal organs in the holothurian *E. fraudatrix* have been well studied^7,12,19–21^. Nevertheless, the successive stages of digestive tube formation are characterized only superficially and are interpreted somewhat differently in various publications^7,19^. Thus, in our work, we conditionally distinguish three stages of anterior gut regeneration, depending on the phases of transdifferentiation of the coelomic epithelial cells.

**Figure 1:**
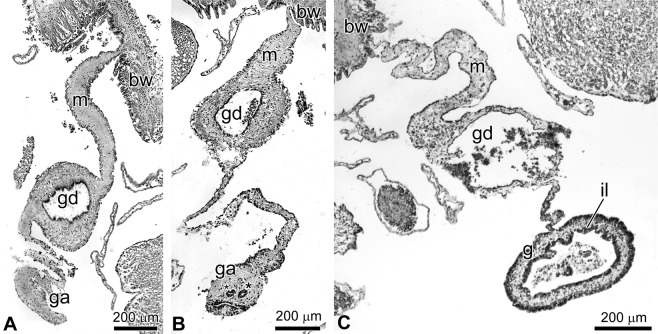
Structure of gut on different stages of regeneration in holothurian *Eupentacta fraudatrix*. (**A**) Connective-tissue thickening on the edge of mesentery on day 3 post-evisceration (first stage). (**B**) Gut anlage on day 6 post-evisceration (second stage); arrow indicates site of migration of coelomic epithelial cells into connective-tissue thickening, asterisks show clusters inside the intestinal anlage. (**C**) Digestive tube on day 12 post-evisceration (third stage). bw – body wall, g – digestive tube, ga – gut anlage, gd – gonoduct, il – intestinal lining, m – mesentery.

The first stage (day 3 post-evisceration) is characterised by formation of thickening on the edge of the mesentery in the anterior part of the holothurians. This thickening represents the intestinal anlage, consisting of connective tissue (Fig. 1А), and is covered by the coelomic epithelial cells that migrate from the mesentery; numerous mesenchymal-like cells are visible inside. During the regeneration of *E. fraudatrix*, the connective-tissue thickening gradually, lengthens and spreads back along the mesentery edge.

The second stage of regeneration (days 5–7 post-evisceration) is characterised by active transdifferentiation of cells. On the ventral side of the anlage, coelomic epithelial cells form folds and migrate into the connective tissue with the formation of numerous cell clusters inside the intestinal anlage (Fig. 1B). During migration, the structure of cells changes, with fragments of tonofilaments and spindle-like structures finally disappearing from their cytoplasm^7,12^. On days 8–9 post-evisceration, microvesicles and secretory vacuoles, characteristic of enterocytes, are found in their apical part^12^. Transforming cells retain their intercellular junctions, i.e., they do not undergo the epithelial-mesenchymal transition^7,12^.

The third stage of regeneration (days 10–12 post-evisceration) is characterised by the presence of well-formed luminal epithelium (Fig. 1C), due to merging of cell clusters. Subsequently, the anterior anlage continues to grow towards the posterior body end along the mesentery edge. The luminal epithelial cells gradually differentiate and acquire a structure characteristic of enterocytes. The unification of anlagen occurs towards the middle of the animal’s body on days 25–27 post-evisceration, with the formation of a continuous digestive tube^7^.

### *De novo* transcriptome assembly

As a result of sequencing of eight libraries, corresponding to the three stages of regeneration and the normal gut of the holothurian *E. fraudatrix*, a total of 413 million (412,999,924) raw paired-end reads were obtained. After filtering and trimming the adapters, 95% paired-end reads were retained with an average quality of 35.7 units by PhredScore 33, and an average read length of 99.4 nucleotides (see Supplementary Table S1). All of these reads, as well as the unpaired reads that remain after filtering and read error corrections, accounted for 5% of the initial number of reads, were used in the assembly. As a preassembly stage, two iterations of read error corrections were performed, reducing the number of paired-end reads. On average, 5% of paired reads are lost. As a result of the assembly, filtering of contaminant and non-coding sequences, and subsequent clustering to identify isoforms, a total of 83,960 transcript isoforms and 70,538 genes were obtained.

In addition, all sequences were removed in which the predicted protein matched the species protein which did not belong to deuterostomes. These sequences satisfied two conditions: the bitscore above 200; the lack of a match with the available protein sequences of *Apostichopus japonicus*, *Parastichopus parvimensis*, *Cladolabes schmeltzii*, *Patiria miniata*, *Lytechinus variegatus*, and *Strongylocentrotus purpuratus*^25–27^ with a bitscore, which was more than 80% of the match bitscore in the nonredundant protein (NRP) NCBI database. We assessed the completeness of the assembly using BUSCO v3^28^. As result, 98.1% of the core metazoan genes (based on 978 core essential genes) and 83% of the single-copy orthologs among them, were identifiable in the transcriptome. Only 21% of reads were mapped back to the assembly. This value is underestimated, due to strict alignment parameters and the presence of only protein coding regions in the assembly.

### Differential expression analysis

**Figure 2:**
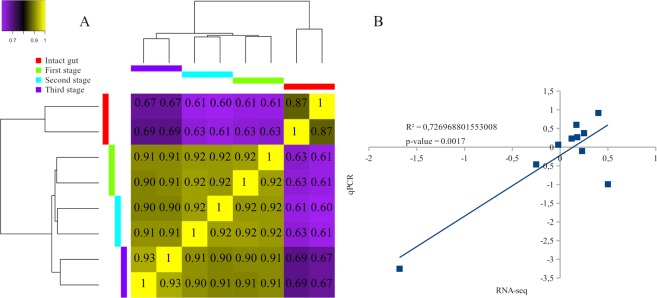
Correlation map of all RNA-seq samples (**A**) and gene expression correlation between RT-qPCR (y-axis) and RNA-seq (x-axis) data. (**B**) The squared Pearson correlation coefficient, p-value, and linear regression line for 5 genes are indicated on plot. (**B**) Each point on the plot (**B**) corresponds to the logarithm (base 2) of the fold change in the second or third stages of regeneration relative to the first stage.

After aligning the reads to assembly and counted mapped events (see Supplementary Table S2), we performed a correlation analysis (Fig. 2A). We found a high correlation of replicates within samples, at almost 91%, while the correlation from the regeneration stages was significantly higher, versus the sample from an intact gut. Validation of expression levels of the 5 genes showed a mean correlation between qPCR and RNA-seq data (Fig. 2B).

**Figure 3:**
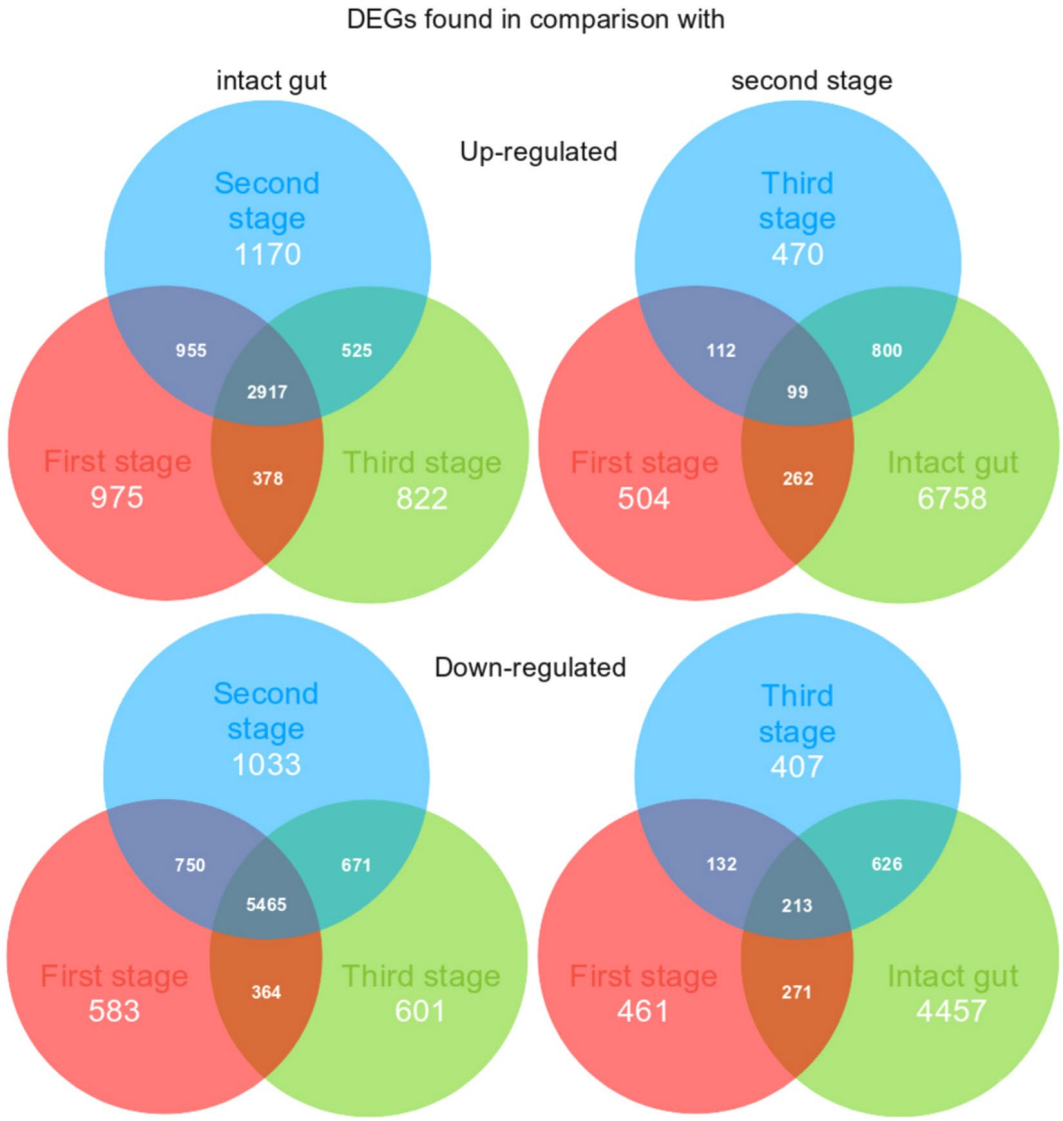
Venn diagrams for up-regulated and down-regulated DEGs.

As a result of the search for DEGs, we found a total of 17,227 and 15,342 genes with notable changes in expression levels for all samples, relative to an intact gut and the second stage, respectively (see Supplementary Data S2). Of these, 13,234 and 11,942 sequences, respectively, had significant hits in the NRP NCBI database (Fig. 3). Thus, most of the DEGs are detected when compared to the three stages of regeneration with the intact sample. The same is evidenced by the map of correlations between samples (Fig. 2A). The observation indicates essential changes in the work of genes after damage and, accordingly, the artificiality of comparing gene expression levels in regenerating and intact tissues. This could probably enable identification of the regulatory genes, whose expression is activated or repressed during regeneration. In this study, the emphasis was on finding the probable transdifferentiation regulators, rather than analysing the entire regeneration process. In this regard, only DEGs obtained in comparison to the second stage will be considered below. In addition, an obvious increase in the number of unique genes in the second stage, when transdifferentiation occurs, is interesting. All of the above only confirms expectations, but is not significant in itself.

### Annotation

The annotation of 83,960 sequences by the BLASTx search against to the NRP NCBI database, resulted in the identification of 48,677 sequences and 37,790 genes with significant hits, including 7,118 sequences having only unnamed hits (see Supplementary Table S3). Hits belonged to 1,022 organisms, and almost 80% of sequence matched echinoderm proteins (see Supplementary Table S4). In addition, there is a high probability for the presence of a few contaminant sequences. However, the lack of a genome for this or any related holothurian species does not allow us to accurately determine the source of these sequences.

The annotation against human and sea urchin proteins led to the identification of 10,358 and 14,617 orthologs, respectively, as we used a new approach to find the best orthologs. This approach allows for the extraction of a larger number of protein–sequence pairs, versus that of the classical reciprocal search^29^. Thus, the reciprocal method provides only 7,791 and 9,292 pairs compared to human and sea urchin proteins, respectively. The difference between our own versus reciprocal methods is that only the best hits are on analysed in the latter. That is, if there is a protein family in an evolutionarily distant species, with a member of one of the species having better alignment with all members of the protein family of an other species, the reciprocal approach enables a match for this member only. In our case, protein pairs were distributed automatically, based on alignment quality, so members of the best pairs were immediately removed from the hit list of all other pairs (algorithm and code described in Supplementary Note).

### Search of the most likely candidates for the role of transdifferentiation regulators

**Figure 4:**
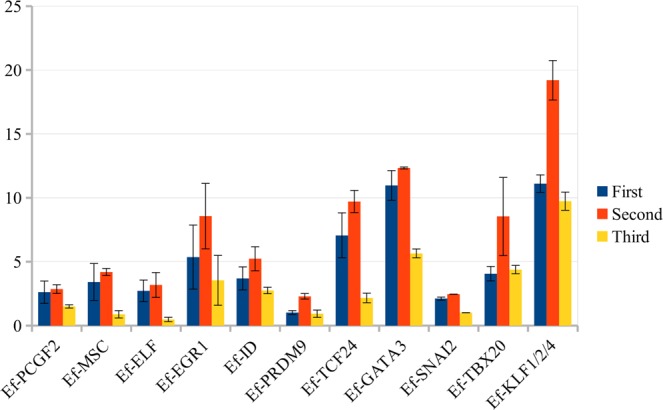
TPM expression values of the most likely candidates for the role of transdifferentiation regulators. All these TFs have a significant peak of expression at second stage of regeneration.

A search for homologs of sea urchin and human TFs resulted in the identification of 308 and 918 TFs, respectively (see Supplementary Table S5). Of these, 265 TF homologs were found among proteins both humans and sea urchin. Given all TFs, 20 were the most interesting in terms of expression in the second stage of regeneration. All homologs were verified using the NRP NCBI database, but for zinc finger TFs were confirmed only matching with zinc finger proteins. Then, 8 putative zinc finger proteins were removed, as none had an unconditional match to known zinc finger proteins. Also, Ef-ELF4 was removed, since the region corresponding to the ETS domain of Ef-ELF2 and Ef-ELF4 is identical, while the rest of the Ef-ELF4 sequence, unlike the Ef-ELF2, has no significant alignment to the NRP NCBI database proteins. The Ef-ELF4 sequence is apparently an assembly error, which is also confirmed by sea urchin data, for which only one *elf* gene was found to be homologous to the human *elf1*, *elf2*, and *elf4* genes^30^. For this reason, by analogy with the sea urchin *elf* gene, we refer below to Ef-ELF2 as Ef-ELF. The expression profiles of the 11 remaining TFs, with TPM values at each stage, are shown in Fig. 4. With the exception of Ef-ID2, they all had a p-value less then 0.05. They belong to 6 TF classes: tryptophan cluster (Ef-ELF), C2H2 zinc finger (Ef-PRDM9, Ef-EGR1, Ef-KLF1/2/4 (KLF2), Ef-SNAI2), bHLH (Ef-TCF24, Ef-MSC, Ef-ID2), C4 zinc finger (Ef-GATA3), polycomb group ring finger (Ef-PCGF2), and T-box (Ef-TBX20).

Ef-ELF (ELF2) belongs to the Ets family, whose members are powerful regulators of cell proliferation, angiogenesis, hematopoiesis, tumour transformation, and differentiation^31,32^. However, the functions of ELF1, ELF2, and ELF4 in chordates are associated with lymphoid and endothelial cell differentiation, hematopoietic stem and progenitor cell survival, and tumour suppression^33–36^. Sea urchin *elf* expression is detected everywhere in late gastrula, being more concentrated in the gut^30^. The latter assumes that the ELF ortholog in echinoderms may be involved in the regulation of the early stages of digestive epithelium formation. Our data agree with this assumption. In *E. fraudatrix*, Ef-elf is expressed at early regeneration stages, during the formation of the gut luminal epithelium (Fig. 4). In the third stage, when the gut tube is already formed, its expression is sharply reduced.

Ef-KLF1/2/4 is probably a homolog of human KLF1, 2, and 4, which is confirmed by the data of Mashanov *et al*.^24^. The KLF proteins play diverse roles in cell proliferation, differentiation, and development^37^. In particular, KLF2 and KLF4 are key TFs that maintain a stem cell-like state and somatic cell reprogramming^38^. In this regard, the expression of Ef-klf1/2/4 in E. fraudatrix may indicate its involvement in the regulation of differentiation and/or transdifferentiation. At all three stages of regeneration, this gene showed a high level of expression. As mentioned above, various transformation processes of the coelomic epithelium occur in this period. On the mesentery surface and lateral sides of the gut tube, myoepithelial cells and peritoneocytes dedifferentiate, migrate, proliferate, and redifferentiate. Their dedifferentiation, embedding in connective tissue, proliferation, and transdifferentiation occur on the ventral side of the anlage (Fig. 1). A two-fold increase in the expression of Ef-klf1/2/4 at the second stage, compared to the first and third stages (Fig. 4) may indicate activation of cell reprogramming and maintenance of the dedifferentiated state during transdifferentiation, when gut epithelium is formed. According to Mashanov *et al*.^24^, expression of klf1/2/4 does not change during regeneration of the intestine and nervous system in the holothurian H. glaberrima. Differences in the dynamics of klf1/2/4 expression between H. glaberrima and E. fraudatrix may reflect different formation mechanisms for intestinal epithelia in these species. In H. glaberrima, intestinal epithelium forms from dedifferentiated cells of the esophagus mucosa, without transdifferentiation^16^.

The expression of the Ef-prdm9 gene is relatively low, compared to the other TFs considered here (Fig. 4). However, its activity at the second stage is more than twice as high as that during other stages. This may suggest its involvement in the regulation of some transdifferentiation aspects. Proteins of the PRDM family are important epigenetic regulators in the development, cell differentiation, and pluripotency in the mouse^39^. In E. fraudatrix, Ef-PRDM9 may be involved in the processes of differentiation and/or maintenance of the dedifferentiated cell state.

EGR1 is involved in the cell cycle progression of various tumour types as well as hepatic regeneration in mammals^40,41^. Recent research has shown that EGR1 is a “putative pioneer factor to directly activate wound-induced genes” in whole-body regeneration of acoels^42^. Ef-EGR1 probably performs similar functions. However, the dynamics of its expression during gut regeneration in E. fraudatrix (Fig. 4) shows that Ef-EGR1 may also play a role in the regulation of transdifferentiation.

SNAI proteins play a crucial role in the epithelial-mesenchymal transition (EMT) and in repressing the mesenchymal-epithelial transition (MET), being important in embryonic and tumour development^43–45^. The co-expression of Ef-snai2 and *Ef-id2* (see below) may indicate partial involvement of the EMT mechanisms in the transformation of coelomic epithelial cells into enterocytes in *E. fraudatrix*. Although transforming cells do not lose contact with neighboring cells^7^, they can acquire some mesenchymal features when migrate to the intestinal anlage, similar to those during the growth of tubular organs^46^. Epithelial cells at the migration tip have a partially mesenchymal character. These cells maintain intercellular junctions, but are flattened and form lamellipodia^47,48^.

To our knowledge, no animal studies on the function of TCF24 in regeneration, development, or carcinogenesis have been carried out. The dynamics of *Ef*-*tcf24* expression in *E. fraudatrix* suggest its involvement in gut regeneration. The expression of *Ef-tcf24* at the second stage is 30 times as high as that of intact individuals (Fig. 4). Moreover, the number of *Ef-tcf24* transcripts reduces abruptly at the third stage, when the embedding of coelomic epithelium and its transformation into gut epithelium is completed. Based on these facts, we can assume this TF to be involved in the regulation of transdifferentiation or dedifferentiation of the coelomic epithelium.

Musculin (MSC) is involved in myogenesis and stem cell functioning in mammals. Its represses myogenesis and acts as a lineage-restricted repressor of embryonic skeletal muscle development^49^. MSC regulates LIF-induced gene expression of some renoprotective factors in the adult kidney-side population cells that are in a pluripotent state^49^. In *E. fraudatrix*, Ef-MSC can perform similar functions. Its relatively low expression (Fig. 4) can be explained by the small number of cells in which *Ef-msc* is activated. Its probably repress the myogenic differentiation of coelomic epithelium cells and maintain their pluripotent state in the formation of digestive epithelium.

Another TF, ID2, is not a transcription factor, despite the presence of the HLH-domain, as it lacks the basic DNA-binding domain. Yet it forms a heterodimer with bHLH TFs, inhibiting their function: in a sea urchin, this is noted as a TF^27^, and its functions and expression seemed noteworthy to us for consideration. ID2 prevents precocious differentiation of epithelial cell progenitors during mouse gut development^50^. Downregulation of ID2 induces dedifferentiation of epithelial cells^51^. Ef-ID2 could perform similar functions during regeneration in *E. fraudatrix*, such as involvement in the dedifferentiation of coelomic epithelial cells and maintenance of their dedifferentiated state. This protein, with Ef-SNAI2, probably regulates cell migration and EMT, similar to their homologues in mammals^52,53^.

The expression of *Ef-pcgf2* during gut regeneration in *E. fraudatrix* is relatively low (Fig. 4), and the difference in its magnitude between stages is negligible. Based on this, we can assume this TF would be involved in the processes that occur throughout the three regeneration stages. It has been shown that PCGF2 is a chromatin-modifying protein that inhibits EMT by downregulation of ZEB1 and ZEB2, in human breast cancer, and negatively regulates stem cell-like properties in human gastric cancer^54,55^. In *E. fraudatrix*, its homolog may be involved in the maintenance and retention of the “mesodermal” properties of coelomic epithelial cells on the gut anlage surface.

Expression of *Ef-gata3* at the first and second stages is almost twice as high as at the third stage, which suggests that this TF is involved in the regulation of gut lining formation. Like its homologs in other animals^56–58^, Ef-GATA3 is apparently involved in the transformation processes of the mesoderm (coelomic epithelium cells).

TBX20 is a crucial cardiogenic TF in mammals^59^. The *tbx20* gene is also expressed in the nervous system and embryonic lateral mesoderm of the mouse^59,60^. It is probable that Ef-TBX20 in *E. fraudatrix* is also involved in the myogenic cell differentiation of the coelomic epithelium and the formation of gut muscles. However, the expression of *Ef-tbx20* at the second stage of regeneration is almost twice as high as that of the first and third stages. In this regard, its participation in the regulation of transdifferentiation can also be assumed.

Obviously, other TFs, which do not show such unambiguous dynamics as those listed above, can participate in the regulation of transdifferentiation. In fact, gene activation can be short-term or done by a small number of cells. In the former case, given sufficiently long intervals between stages, we could not record any sharp variation in expression. In the latter case, due to the use of the whole anlage, rather than single cells or equal cell numbers, the average number of transcripts of such genes may be small, so there may be no difference between certain stages of regeneration. Among these genes, a homolog of human s*ox17* deserves special attention. In vertebrates, it is a marker of the formation of the endoderm and gut^61,62^. According to Campbell *et al*.^23^, SOX17 is involved in the transdifferentiation of muscle cells into enterocytes in zebrafish. In *E. fraudatrix*, the s*ox17* homolog is expressed as early as the first stage of regeneration, and subsequently the number of its transcripts increases (see Supplementary Table S5). This could indicate that the initial stages of determination of enterocyte precursors are launched on the surface of the anlage, before the cells migrate to the connective tissue.

### Network of enrichment biological processes and pathways

**Figure 5:**
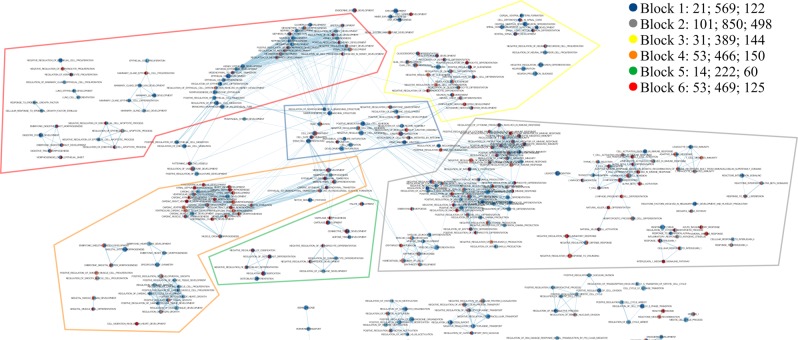
Network of enrichment biological processes and pathways associated with the most likely candidates for the role of transdifferentiation regulators. Nodes represent biological process (gene set). Edges represent overlap between pair of gene sets. Node size and edge width depend on the number of genes. Node fill represents enrichment scores of terms at the second stage of regeneration relative to the first (right half) and third stages (left half), respectively. The color gradient represents an increase (red color) or decrease (blue color) in the level of expression (depend on the enrichment score) in the second stage, relative to the first or third. A block description is a block number, the number of terms, and the number of all and unique genes in a block. The full version of the network in Cytoscape format is represented in the Supplementary Data S1.

By constructing a network associated with 11 TFs listed above, we obtained 490 enrichment pathways and biological processes, combining 3,168 homologs of human proteins (Fig. 5). Of them, 204 and 71 Gene Ontology (GO) terms had down- and upregulated genes, respectively, at the second stage relative to both the first and third stages. The obtained network primarily attracts attention by several blocks of closely interrelated processes associated with morphogenesis, confirming the involvement of the TFs we revealed during gut regeneration in E. fraudatrix.

The central part is occupied by terms, regarding the regulation of morphogenesis, intercellular interactions, and interaction of cells with the extracellular matrix (block 1). Of interest is the increase in expression of the genes involved in cell fate determination and commitment, over three stages. Indeed, in E. fraudatrix, the intestinal and coelomic epithelia form and their cells specialize in this period (days 3–11 post-evisceration)^7,12^. Thus, in this block, genes that trigger or control the fate commitment change of coelomic epithelial cells.

The second group includes terms related to hematopoiesis and immune response. The expression of a large number of immune-related genes may indicate postinjury activation of protective functions in E. fraudatrix. This is not surprising, as the animals must protect the internal environment from pathogens that can penetrate from seawater during evisceration.

Terms related to neurogenesis in the third group is also logical, as the coelomic epithelium of echinoderms contains a large number of nerve processes and neurons that form the basiepithelial nerve plexus. During the digestive system’s regeneration of E. fraudatrix, this plexus forms again in the coelomic epithelium of the intestine.

The fourth group contains processes related to cardiac development, mesenchymal morphogenesis, and EMT. Although echinoderms lack a heart, this group is noteworthy, as most processes in it are characterised by increased gene expression at the second stage of regeneration. An analysis of the GO terms available shows they are associated with processes of mesodermal cells reorganization, such as migration, proliferation, and EMT, occurring in heart development of mammals^63^. In E. fraudatrix, similar processes occur in coelomic epithelium, which is transformed during intestinal regeneration. Most genes associated with EMT have a decreasing expression of dynamics, with a maximum at the first stage of regeneration. Activation of EMT mechanisms during gut regeneration in E. fraudatrix is unusual and to some extent contradicts morphological data; the intercellular junctions are not destroyed during formation of intestinal epithelium, and epithelial cells are not transformed into mesenchymal cells. This is explained by the conversion process of part of the coelomic epithelium into mesenchyme, which is not associated with the enterocyte formation, as described by the holothurian H. glaberrima^64^. In addition, when coelomic epithelium is immersed in the connective-tissue anlage of the intestine in E. fraudatrix, cells are likely to acquire some mesenchymal features, as mentioned^46–48^. It is likely that partial and full EMT are regulated by the same genes.

The fifth block contains the GO terms associated with extracellular matrix remodelling, and the differentiation of chondrocytes and osteoblasts. Gene expression data are consistent with morphological events occurring during the intestinal regeneration of E. fraudatrix. The formation of the connective tissue anlage of the gut occurs in the early stages post-evisceration, manifested as higher gene activity in the first stage. The terms associated with the differentiation of chondrocytes and osteoblasts possibly reflect the processes of fibroblast changes, as well as the active formation of extracellular matrix of the gut wall.

The sixth block is quite large and contains GO terms associated with kidney development and epithelium morphogenesis. In the regeneration in E. fraudatrix, it should be considered as a group of genes involved in epithelial transformation, since holothurians lack kidneys, or any specialized excretory organs^22^. In E. fraudatrix, homologs of these genes are apparently involved in the reorganization of migrating clusters of epithelial cells into the tubular luminal intestinal epithelium – as shown by the presence of GO terms, such as “nephron tubule formation,” “mesonephric tubule morphogenesis,” etc. In the context of this article, genes of the sixth block are less interesting, being associated with processes of morphological reorganization of epithelium, not with transdifferentiation.

In any case, due to the difficulty of interpreting the processes identified for humans, the main function of this analysis was to obtain evidence of the involvement of the TF set in proliferation, cell differentiation, and embryonic development. This consideration has been formed as a function of two factors. First, it is impossible to find an ortholog for every human gene in the E. fraudatrix genome. The number of genes and more important, the composition of gene families is too different between such evolutionarily distant organisms. At best, we may consider one gene homologous to several human genes. In this regard, the exact function of genes cannot be predicted by human homologs. Second, human biological processes cannot be directly applied to evolutionary and structurally distant organisms. Thus, we consider it critically important to avoid proposing definite hypotheses about the functions of proteins and processes, based only on the homology and functional annotation of evolutionarily distant species. In other words, any analysis based on homology must be interpreted carefully.

## Conclusions

Our study shows that the regeneration of the digestive system in *E. fraudatrix* causes a significant change in the expression of many genes, including TFs. Among the latter, 11 can be distinguished, the expression of which increases on days 5–7 post-evisceration. In this period, coelomic epithelium is immersed in the connective tissue of the intestinal anlage, with the coelomic epithelial cells transforming into enterocytes. We suggest that the 11 above-noted TFs as the most likely candidates for the role of transdifferentiation regulators in holothurians. To continue with the study on the mechanisms of transdifferentiation, it will be necessary to identify cells in which these TFs are expressed, and how gene cascades are triggered by each TF.

## Methods

### Animals

Adult mature individuals of the holothurian *Eupentacta fraudatrix* (D’yakonov *et al*., 1958) were collected in the Peter the Great Bay, Sea of Japan, and kept in 3 m^3^ tanks during one week with running aerated seawater at 16 °C. Evisceration was induced by injection of 2% KCl. Then holothurians were transferred to 370-liter tanks with aerated seawater at 20–22 °C, where they regenerated their internal organs.

### Sample collection and RNA extraction

Tissue of gut was sampled from the intact gut and on the third (first stage of regeneration), fifth–seventh (second stage), and tenth (third stage) days post-evisceration. A total of 10 individuals were selected from each of the stages. Each sample was represented in two biological replicates with 5 individuals, pooled together, per replicate. Fixation was performed in 3 mL RNAlater (Sigma, USA) for 24 h at 4 °C; then the material in RNAlater was stored at −20 °C. Before isolating total RNA, the tissue was precipitated in sterile seawater. Homogenization was carried out in ExtractRNA (Evrogen, Russia) with metal balls on a TissueLyser LT homogenizer (Quagen, Germany). Total RNA was isolated using phenol-chloroform extraction^65^.

### Transcriptome sequencing

All steps for preparing the libraries and sequencing were carried out at the Evrogen company (Evrogen, Russia). The libraries were prepared using a TruSeq Stranded mRNA Library Prep Kit (Illumina, USA), and fragments with a length of 250–450 nucleotides, including adapters, were selected. After testing the quality on an Agilent TapeStation system, paired-end sequencing (2 × 100) was performed on an Illumina HiSeq. 2500 sequencing system. Raw reads were loaded to the SRA NCBI database with the accession numbers from SRR8297983 to SRR8297990 for the three stages of regeneration and intact gut, respectively.

### *De novo* transcriptome assembly

Raw reads from eight libraries (listed in Supplementary Table S1), obtained by us in FASTQ format, were processed using Trimmomatic 0.36 software^66^ with “LEADING:20 TRAILING:20 SLIDINGWINDOW:5:20 AVGQUAL:25 MINLEN:21” parameters to achieve clean reads by removing those containing adapter sequences, poly-N sequences, or low-quality bases.

Then the clean reads were corrected and assembled using SPAdes 3.13 software^67^ with 2 iterations for read correction step and with a k-mer length of 25, 33, and 49. Of all the obtained contigs, the Coding Sequences (CDSs) with a minimum length of 30 amino acid residues were extracted using TransDecoder 5.5.0 software^68^. The code of TransDecoder was modified in such a way that stop-codon or the beginning of the sequence, but not “ATG” (Met) or the beginning of the sequence, was taken for the beginning of CDS. All CDSs were verified using BLAST-search^69^ by the database containing protein sequences from the SwissProt (11.12.18) and the Echinobase (11.12.18) databases^27,70^, as is described in the manual to TransDecoder.

Subsequently, the obtained sequences were assembled with HomoloCAP script as described in the article^26^. The resulting sequences were filtered according to the NCBI requirements and uploaded to the TSA NCBI Database with the index GHCL00000000. Transcriptome completeness was assessed using BUSCO v3^28^ in mode “protein” with a Metazoa v9 dataset.

### Differential expression analysis

To find DEGs, the number of mapped reads was calculated in RSEM v1.3.1 software^71^; paired-end reads were aligned in Bowtie 2.3.4 software^72^ with the following parameters: “–no-mixed–no-discordant–gbar 50–end-to-end -k 200–very-sensitive–minins 50 -q–maxins 450–fr”. The procedure of detection of significant changes in expression was modified. Thus, for analysis we used sequences satisfying the following conditions: the total number of mapped reads was more than 50; more than tenfold coverage; a zero number of mapped reads allowed at any stage. After this filtration, differential expression was evaluated for the sequences in DESeq. 2 v1.18 software^73^ (see Supplementary Data S2). The analysis was performed in two variants: in the first case, the control sample was the first stage of regeneration; in the second case, intact gut. Those DEGs, whose expression level was two times as high at sample than at the control, and with the p-value less than 0.05, were considered actual.

### Validation of gene expression

In order to validate the changes in gene expression determined by RNA-seq, we selected 5 genes of TFs for qPCR analysis. Poly(A) RNA was extracted as described above, but from other individuals than used for sequencing. RNA was isolated independently three times (5 individuals per repeat) for each of the three stages of regeneration. Sequences of PCR primers are shown in the Supplementary Table S6. RNA was reverse transcribed with poly-dT primers as described in the protocol of MMLV RT kit (Evrogen, Russia). PCR reaction was performed using a qPCRmix-HS SYBR kit (Evrogen, Russia) according to the manufacturer’s protocol for CFX96 Touch system (Bio-Rad, USA). The reactions were performed on three independent RNA samples per condition. Each sample was analyzed at least twice, making sure that the difference between technical replicates was lower than 0.5 Ct. The first regeneration stage was used as a control sample. All the expression values were normalized relative to the Ct geometric mean of Tubulin and EF1a genes. The delta-delta Ct method was used to evaluate expression. Primer efficacy was evaluated using ten serial dilutions (1:2) of cDNA combined from all samples in CFX Manager v3.1 software (Bio-Rad, USA). Specificity of the primers was determined by Sanger sequencing of target amplicon.

### Annotation

Annotation was carried out by several protein databases with the standard e-value for BLASTP 2.7.0, which was 1e-5. Basic annotation was by the NR NCBI Database (11.12.18); the annotation for enrichment analysis was performed by BLASTP searching against human proteins from the Ensemble database v95^74^; the annotation for finding the transcription factors was based on the human proteins from Ensemble and sea urchin proteins from the Echinobase project^27^. Only TF homologs with a predicted protein length of more than 200 amino acid residues were taken into consideration. Orthologs of human proteins were identified using a custom Python script that implements modified reciprocal method for finding the best hit (algorithm and code described in Supplementary Note).

To identify the most likely candidates for the role of transdifferentiation regulators, only the TFs satisfying the three conditions were taken into consideration: the values of TPMs (Transcripts Per Kilobase Million) of the second stage higher than unity; the average value of LogFC (logarithm of fold change with base 2) of the second stage relative to the first and third stages more than half; the value of LogFC (logarithm of fold change) of the second stage relative to the first or third stages more than unity.

The enrichment analysis of biological processes and pathways was performed with GSEA software^75^ in accordance with the EnrichmentMap protocol for RNA-seq data^76^. The gene sets for biological processes and pathways were accessed from the MsigDB database v6.2^75^. Only processes or pathways containing at least one of the 11 TFs selected as described in the results section were used. Then, results of the enrichment analysis were visualized using EnrichmentMap plug-ins in Cytoscape software^77^ (see Supplementary Data S1).

## Supplementary information

Supplementary Info.

Table S1.

Table S2.

Table S3.

Table S4.

Table S5.

Table S6.

Supplementary Note.

Data S1.

Data S2.

## Data Availability

The raw reads, obtained by us, were uploaded to the SRA NCBI Database with the accession numbers from SRR8297983 to SRR8297990 for the three stages of regeneration and the normal gut, respectively. The assembly was uploaded to the TSA NCBI Database with the index GHCL00000000.
