# Supplementary material for: The Eupentacta fraudatrix transcriptome provides insights into regulation of cell transdifferentiation
Source: Sci Rep. 2020 Jan 30;10:1522. doi: 10.1038/s41598-020-58470-0 (PMC6992634; doi:10.1038/s41598-020-58470-0)
Supplement: Supplementary file 1 — Supplementary Info. [file 41598_2020_58470_MOESM1_ESM.doc]

**The *Eupentacta fraudatrix* transcriptome provides insights into regulation of cell transdifferentiation**

Alexey V. Boyko^1*^, Alexander S. Girich^1^, Ekaterina S. Tkacheva^1^, Igor Yu. Dolmatov^1,2^

**Table S1. The basic features of analyzed Illumina paired-end libraries before and after filtration**

**Table S2. Number of mapped reads per sequence in three stages of intestinal regeneration and normal gut.**

**Table S3. Results of BLASTp searching of best hits against NCBI protein non-redundant database.**

**Table S4. Taxonomic distribution of hits in Protein non-redundant NCBI database**

**Table S5. List of homologs of sea urchin and human transcription factors with TPM values.**

**Table S6. List of primers used for qPCR**

**Data S1. The full version of the network in Cytoscape format.**

**Data S2. The DESeq2 results of the evaluation of differential expression relative to the second stage of regeneration (DESeq2.2s) and relative to the intact gut (DESeq2.Intact).**

**Note. The description of the BLAST result analysis algorithm and script code.**
