# Supplementary material for: The Eupentacta fraudatrix transcriptome provides insights into regulation of cell transdifferentiation
Source: Sci Rep. 2020 Jan 30;10:1522. doi: 10.1038/s41598-020-58470-0 (PMC6992634; doi:10.1038/s41598-020-58470-0)
Supplement: Supplementary file 8 — Supplementary Note. [file 41598_2020_58470_MOESM8_ESM.zip › README.doc]

**The *Eupentacta fraudatrix* transcriptome provides insights into regulation of cell transdifferentiation**

Alexey V. Boyko^1*^, Alexander S. Girich^1^, Ekaterina S. Tkacheva^1^, Igor Yu. Dolmatov^1,2^

The script accepts an input file with the blast results in a table format, column numbers with qseqid, sseqid and bitscore. The script works in several steps and the result is output to the input_file.best file.

1. Reading file. Compilation of three dictionaries. The first (full_dict) has keys from sseqid, the value is a list that includes all the data for sseqid in the form of lists from bitscore, qseqid and full line from input_file. The second (sseqids_dict) has keys from sseqid, the value is a list that includes all the data for sseqid in the form of lists from bitscore and qseqid. The third (qseqids_dict) has keys from qseqid, the value is a list that includes all the data for qseqid in the form of lists from bitscore and sseqid.
2. Sort the values of all dictionaries. Removing from qseqids_dict and sseqids_dict all values that have bitscore less than the best for the key (qseqid or sseqid) bitscore * 0.6. Removing from full_dict all values that have bitscore less than the best for the key (sseqid) bitscore * 0.5.
3. Iterative traversal of the sseqids_dict dictionary so far has at least one sseqid for which the number of entries in the dictionary is greater than 1. Three conditions are checked during the traversal.
   3.1 If there are no entries for sseqid, then all entries that have sseqid in the qseqids_dict dictionary are deleted.
   3.2 If the best qseqid corresponding to sseqid in sseqids_dict does not have any entries in qseqids_dict, then all entries having qseqid in sseqids_dict are deleted. Then condition 1 applies.
   3.3 If the best qseqid corresponding to sseqid in sseqids_dict has the same best sseqid in qseqids_dict, then the sseqid:qseqid pair is saved and all entries from qseqids_dict and sseqids_dict that have such sseqid or qseqid are deleted.
4. Using the values identified in point 3.3, pairs and data from the full_dict dictionary, complete records from the input_file are restored and written to input-file.best.
